# Calprotectin and Lactoferrin Faecal Levels in Patients with *Clostridium difficile* Infection (CDI): A Prospective Cohort Study

**DOI:** 10.1371/journal.pone.0106118

**Published:** 2014-08-29

**Authors:** Andrew Swale, Fabio Miyajima, Paul Roberts, Amanda Hall, Margaret Little, Mike B. J. Beadsworth, Nick J. Beeching, Ruwanthi Kolamunnage-Dona, Chris M. Parry, Munir Pirmohamed

**Affiliations:** 1 The Wolfson Centre for Personalised Medicine, University of Liverpool, Liverpool, United Kingdom; 2 The Royal Liverpool and Broadgreen University Hospital Trust, Liverpool, United Kingdom; 3 Institute of Infection and Global Heath, University of Liverpool, Liverpool, United Kingdom; 4 NIHR Health Protection Unit in Gastrointestinal Infections, Liverpool, United Kingdom; 5 Liverpool School of Tropical Medicine, Liverpool, United Kingdom; 6 Department of Biostatistics, University of Liverpool, Liverpool, United Kingdom; Institute Pasteur, France

## Abstract

Measurement of both calprotectin and lactoferrin in faeces has successfully been used to discriminate between functional and inflammatory bowel conditions, but evidence is limited for *Clostridium difficile* infection (CDI). We prospectively recruited a cohort of 164 CDI cases and 52 controls with antibiotic-associated diarrhoea (AAD). Information on disease severity, duration of symptoms, 30-day mortality and 90-day recurrence as markers of complicated CDI were recorded. Specimens were subject to microbiological culture and PCR-ribotyping. Levels of faecal calprotectin (FC) and lactoferrin (FL) were measured by ELISA. Statistical analysis was conducted using percentile categorisation. ROC curve analysis was employed to determine optimal cut-off values. Both markers were highly correlated with each other (r^2^ = 0.74) and elevated in cases compared to controls (p<0.0001; ROC>0.85), although we observed a large amount of variability across both groups. The optimal case-control cut-off point was 148 mg/kg for FC and 8.1 ng/µl for FL. Median values for FL in CDI cases were significantly greater in patients suffering from severe disease compared to non-severe disease (104.6 vs. 40.1 ng/µl, p = 0.02), but were not significant for FC (969.3 vs. 512.7 mg/kg, p = 0.09). Neither marker was associated with 90-day recurrence, prolonged CDI symptoms, positive culture results and colonisation by ribotype 027. Both FC and FL distinguished between CDI cases and AAD controls. Although FL was associated with disease severity in CDI patients, this showed high inter-individual variability and was an isolated finding. Thus, FC and FL are unlikely to be useful as biomarkers of complicated CDI disease.

## Introduction

*Clostridium difficile* infection (CDI) is a major cause of nosocomial infections in patients undergoing antimicrobial treatment and is subject to mandatory notification in the UK [1]–[3]. It accounts for approximately 20% of antibiotic-associated diarrhoea cases. CDI pathogenesis is attributed to the action of two potent toxins, A and B [4], [5]. Their synergistic effects cause fluid accumulation and damage to the epithelial mucosa [6], further eliciting pro-inflammatory cytokine release [7], [8]. Concurrent activation and recruitment of neutrophils results in an inflammatory response in the gastrointestinal tract of CDI patients, but this is variable, ranging from self-contained mild inflammation to severe pseudomembranous colitis [9], [10].

Toxins are the essential virulence factors accounting for CDI pathogenicity. Current diagnostic tools rely on their detection by either cytotoxin neutralisation or enzyme immunoassays. Multi-step algorithms have also been adopted in an attempt to improve sensitivity by combining toxin detection with sensitive screening of the presence of the organism by selective culture, glutamate dehydrogenase antigen (GDH) detection and/or nucleic acid amplification tests (NAAT) of the locus of pathogenicity (PaLoc) [11]. These tests do not allow for stratification of disease severity and prognosis in patients with CDI. Validated non-invasive enteric markers for CDI that allow for better patient assessment and enable a more personalised approach to treatment would be valuable [11].

Faecal material represents a very complex and heterogeneous biological matrix. Candidate faecal biomarkers must possess properties that ensure reliability and reproducibility of results and they must be unaffected by extra-digestive processes. Faecal calprotectin (FC) and faecal lactoferrin (FL), derived predominantly from activated neutrophils have both been extensively evaluated in inflammatory bowel disease (IBD) and infectious diarrhoea [12]–[22].

FC and FL have also been evaluated in CDI in a small number of studies (Table 1). Some have shown an association of FC in several acute diarrhoeal diseases caused by bacteria, with the highest mean levels observed in patients with CDI (192 mg/L) [23]. Others have shown a significant association when comparing FC levels in toxin positive and GDH positive plus tcdA/tcdB PCR confirmed patients when compared to diarrhoea controls [24]. Similarly, FL has also been shown to be elevated in CDI patients [20], [25]–[27], with more recent studies suggesting a positive correlation with disease severity [28]–[30] and fluoroquinolone resistance [31]. There are however limitations with the published studies: these include their retrospective nature, limited phenotype data, lack of matched controls, use of non-quantitative tests, and variations in the assessment of CDI outcome measures. Sample sizes have varied from 2 to 87, and none of the studies have compared FC and FL in the same patient groups. In this study, we use a prospective design, a carefully phenotyped cohort and simultaneous evaluation of both faecal markers, to investigate whether these faecal biomarkers would have clinical value in patients with CDI.

**Table 1:** Overview of previous studies evaluating the role of lactoferrin and calprotectin in faeces in patients with *Clostridium difficile* infection.

Study	Country	Healthcare setting	Paticipants	Measure used	Results	Associated outcomes (p-value)
**Faecal Lactoferrin**						
Steiner et al. (1997)^30^	USA	Hospital	Mild CDI (n = 6)	Qualitative (positive/negative)	1/6 = positive	Disease severity* (P = 0.021)
			Severe CDI (n = 12)		9/12 = positive	
Vaishnavi et al. (2000)^27^	India	Hospital	CDI cases (n = 41)	Qualitative (positive/negative)	33/41 = positive	*C. diff* toxin positivity & negativity (P<0.001 for both)
			Diarrhoea controls (n = 190)		123/190 = negative	
Pawlowski et al. (2009)^31^	USA	Hospital	CDI cases (n = 34)	Cut-off (72.5 µg/g)	10 resistant = >72.5 µg/g	Moxifloxacin resistance (P = 0.041)
					16 resistant = <72.5 µg/g	
					8 susceptible = <72.5 µg/g	
Archbald-Pannone et al. (2010)^26^	USA	LTCF	CDI cases (n = 2)	Continuous	134.1 µg/ml	*C. diff* colonisation (P = 0.008)
			Diarrhoea controls (n = 22)		28.8 µg/ml	
Van Langenberg et al. (2010)^20^	Australia	Hospital	CDI cases (n = 8)	Continuous	33.3 µg/ml	*C. diff* positivity (P = 0.017)
			Diarrhoea controls (n = 334)		22.6 µg/ml	
El Feghaly et al. (2013)^28^	USA	Hospital	CDI cases (n = 120)	Cut-off (7.25 µg/ml)	72/120 (60%) = >7.25 µg/ml (Outcome data not provided)	Severe HINES VA Score** (P = 0.002)
Boone et al. (2013)^29^	USA	Hospital & outpatients	Mild CDI (n = 7)	Continuous	73 µg/g	Disease severity*** and Ribotype 027 (P = 0.0003 & P = 0.012)
			Moderate CDI (n = 57)		292 µg/g	
			Severe CDI (n = 21)		961 µg/g	
LaSala et al. (2013)^25^	USA	Hospital	GDH neg (n = 43)	Continuous	13 µg/ml	Toxin positivity vs. GDH negative; p = 0.006
			GDH positive/Tox neg/PCR neg (n = 14)		18 µg/ml	Toxin positivity vs. GDH positive/CDT negative/PCR negative; p = 0.002
			GDH positive/Tox neg/PCR positive (n = 30)		80 µg/ml	Toxin positivity vs. GHD positive/CDT negative/PCR positive; p = 0.015
			GDH positive/Tox positive (n = 25)		24 µg/ml	-
Shastri et al. (2008)^23^	Germany	Hospital	CDI cases (n = 87)	Continuous	192 mg/l	-
			Healthy controls (n = 200)		171/196 = <15 mg/l	
Whitehead et al. (2014)^24^	UK	Hospital	Tox positive (n = 45)	Continuous	336 µg g-1	*C. diff* positivity (P<0.05)
			GDH positive/PCR positive (n = 75)		249 µg g-1	
			Diarrhoea controls (n = 99)		106 µg g-1	

*Disease was considered severe if any of the following was present: diarrhoea severe enough to produce clinical signs of volume depletion and to require hospitalization, WBC count of >10,000/ml, or temperature of >38.3°C.

**Scoring system accounting for fever (>38°C), ileus (clinical or radiographic), systolic blood pressure (<100 mmHg), WBC (15000<WBC<30000 cells/µl) and CT scan findings (colonic wall thickening, colonic dilatation, ascites).

***Automatically classified as severe if age ≥65 years, WBC>15×10^9^/L, stool ≥10 per day, not able to tolerate oral intake, usually abdominal complaints, radiographic or peritoneal signs, multiple comorbidities including but not limited to renal failure and immunosuppression.

## Methods

### Cohort

A cohort of 216 patients was recruited from a large hospital setting in Merseyside, UK. Consecutive patients with healthcare-associated diarrhoea, which was defined as ≥3 liquid stools passed per day in the 24 hours preceding assessment, an onset after being in hospital for over 48 hours and recent exposure to either antimicrobials and/or proton pump inhibitors, were eligible for inclusion. Relevant information on demographics, admission and clinical evaluation was collected for each patient who consented to participate and recorded into an anonymised case report proforma.

Blood and faecal specimens were collected from patients at study entry. Faecal samples were analysed by *Clostridium difficile* toxin (CDT) testing using a commercial TOX A/B II ELISA kit (Techlab, Blacksburg, USA) and selective anaerobic culture for 48 h using Brazier’s cefoxitin-cycloserine egg yolk agar (CCEY) (Lab M Ltd, Bury, UK). Isolates were identified by characteristic smell, colonial morphology and fluorescence under long wave UV light. Identification was confirmed using a latex agglutination test for *C. difficile* somatic antigen (Oxoid, Basingstoke, UK). Isolates were stored on PROTECT beads (Technical Services Consultants Ltd, Heywood, UK) at −70°C. PCR ribotyping of isolates was performed using a standard method [32] and compared to a library of circulating nosocomial strains [33]. A multiplex PCR assay targeting a species-specific internal fragment of the triose phosphate isomerase (tpi) housekeeping gene, internal core sequences of both toxins A (tcdA) and B (tcdB) genes was used to confirm all isolates as *C. difficile* and verify their individual toxigenicity [34]. The initial diagnosis of CDI was made by the responsible clinical teams, and the research team only became involved when a diagnosis had been confirmed by a *C. difficile* toxin positive ELISA result. Using these criteria, there were 164 CDI cases. Control patients (n = 52) were individuals with evidence of antibiotic-associated diarrhoea (AAD), negative toxin ELISA test and microbiological culture, with no past history of CDI.

The severity of CDI symptoms was assessed at baseline by research nurses as per guidelines proposed by Public Health England – this is based on white blood cell count (>15×10^9^/L), acutely rising blood creatinine (>50% increase above baseline), fever (temperature >38.5°C), evidence of severe colitis (abdominal signs, radiology) and further complications such as hypotension, partial/complete ileus, toxic megacolon and colectomy [35]. We have further adjusted the white blood cell count to a more stringent cut-off of >20×10^9^/L. Duration of symptoms from the diarrhoea start date was recorded and then dichotomised into episodes lasting more or less than 10 days, while mortality was actively monitored for a period of 30 days. Recurrent CDI was defined as the development of subsequent CDI episodes up to a period of 90 days following treatment of the initial episode. These different parameters collectively represent complicated CDI disease.

### Ethics statement

Ethical approval for the study was obtained from the Liverpool Research Ethics Committee under reference numbers 08/H1005/32 and each patient provided written informed consent prior to recruitment.

### Biomarker measurement in stools

Both FC and FL levels were measured using commercially available IVD ELISA kits (Calpro, Lysaker, Norway; IBD Scan Techlab, Blacksburg, USA, respectively). All procedures were carried out according to manufacturer’s instructions, with the exception of the FL sample preparation step, whereby an inoculation loop was used as an agitator during a 30 minute shaking step in order to ensure optimal recovery of proteins. Where necessary, further dilutions and extra points on the standard curve were included. A standard 4-parameter logistic nonlinear regression method was used to calculate faecal biomarker concentrations.

### Statistical analysis

Levels of FC and FL were subject to a 4-tier percentile categorization (i.e. Low <25%, Medium 25–50%, High 50–75% and Very High >75%). Univariate binary logistic regression was conducted for both case-control comparison and sub-group analysis of cases for the outcomes proposed above. Covariates including age, gender, BMI, score on Charlson Comorbidity Index, presence of ribotype 027 and time delay between testing positive and subsequent recruitment were assessed. As age was already included as an individual covariate, we calculated our Charlson Comorbidity Index unadjusted for age, consistent with previous studies [36]–[38], in order to avoid introducing an undesirable level of collinearity into our analysis. Statistically significant covariates were added to the final regression model to produce adjusted p-values and ORs. A p-value of <0.05 was considered significant. Power calculations were simulated using nQuery Advisor + nTerim 2.0 (Statistical Solutions Ltd., Cork, Ireland).

ROC curve analysis was conducted to identify optimal cut-off values for our cohort and to compare these against the recommended kit values established for active intestinal inflammation. The Pearson correlation coefficient was employed to assess the relationship between the faecal markers.

## Results

### Demographics

Demographics of the patient cohort are summarised in Table 2. No significant differences were observed between CDI cases and AAD controls for mean age (70.2 yrs versus 66.4 yrs; P = 0.13), gender (58% female versus 67% female, respectively; P = 0.26) or median Charlson Comorbidity Index score (1.0 versus 1.0; P = 0.22). However, significant differences were identified for mean BMI (24.6 versus 28.2; P<0.01) and the median time delay between testing positive and subsequent recruitment (3.0 days versus 2.0 days; P<0.01). *C. difficile* isolates were successfully recovered from 149 (91%) of the CDI cases, of which all were toxigenic and 72 (48%) had the ribotype 027/NAP1.

**Table 2:** Demographics of the patient cohort.

	CDI Cases (n = 164)	AAD Controls (n = 52)	P-value*
**Patient’s characteristics**			
Gender – Female n (%)	95 (58)	35 (67)	0.26
Age – Mean in years (SD)	70.2 (15.9)	66.4 (15.8)	0.13
BMI – Mean (SD)	24.6 (6.4)	28.2 (6.9)	<0.01
Charlson Comorbidity Score** – Median (IQR)	1.0 (0.0–2.0)	1.0 (0.0–2.0)	0.22
Time delay prior to recruitment, days – Median (IQR)	3.0 (2.0–4.8)	2.0 (2.0–3.0)	<0.01
**Clinical Parameters**			
All cause death within 30 days – n (%)	14/164 (8.5)	1/52 (1.9)	0.13
Duration of symptoms - 10 days and over – n (%)	83/145^a^ (57.2)	12/46^a^ (26.1)	<0.01
Severity at baseline – n (%)	48/164 (29.3)	-	-
Recurrence within 90 days – n (%)	53/116^b^ (45.7)	-	-
Frequency of ribotype 027– n (%)	72/149^c^ (48.3)	-	-

*n: number; CDI: Clostridium difficile infection; AAD: Antibiotic-associated diarrhoea; BMI: Body mass index; IQR: Interquartile range; SD: Standard deviation;*

*Means for normally distributed, continuous variables were compared using Independent samples T-test for continuous, for non-normal distribution median values were compared using Mann Whitney U test. Categorical data was assessed using a Chi-squared test for all counts >5, and Fisher’s Exact test for those <5;

**Charlson comorbidity score is calculated without accounting for age (see statistical methods);

a *Data regarding duration of symptoms was unavailable for 19 of our cases and 6 of our controls;*

b *Data regarding recurrence of disease within 90 days was unavailable for 48 of our cases;*

c *Isolates were successfully recovered from 149/164 cases and thus ribotyping could not be done in 15 of our cases.*

The proportion of patients suffering from symptoms of 10 or more days was higher amongst CDI cases compared with controls (57.2% versus 26.1%, p<0.01). Of the CDI cases, 29.3% (48/164) were assessed as having severe disease, while 46% (53/116) of cases experienced recurrent episodes during the 3-month follow-up period.

### Power calculations

For both biomarkers, power to detect a significant difference was calculated as ≥97% for the majority of analyses (Table S1). However, for analysis of 30-day mortality for both FL and FC and prolonged symptoms for FL, we had inadequate power.

### Faecal concentrations of FC and FL in CDI

**Figure 1:**
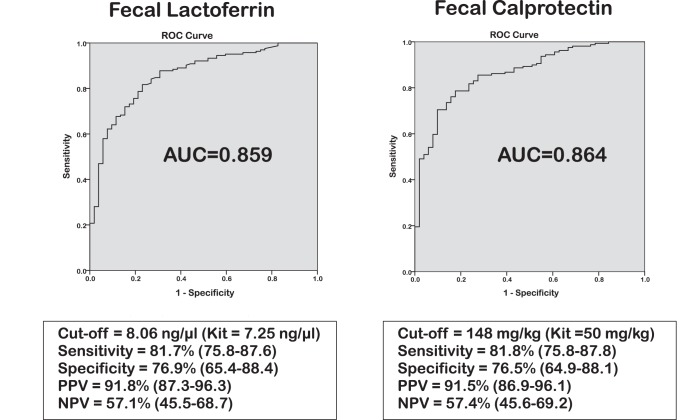
ROC curve analyses of Faecal Lactoferrin and Faecal Calprotectin concentrations in *Clostridium difficile* infection cases (n = 164) versus Antibiotic-associated diarrhoea controls (n = 52).

Median levels of both FL and FC were significantly higher in CDI cases compared to AAD controls (Tables 3 & 4). This was confirmed by percentile case-control analysis [Tables 3 & 4; p-value <0.0001 for both]. ROC case-control analysis of FL resulted in a cut-off value of 8.1 ng/µl with an AUC of 0.86 (95% CI 0.80–0.92), producing a sensitivity of 81.7% (95% CI 75.8–87.6%), specificity of 76.9% (65.4–88.4%), positive predictive value (PPV) of 91.8% (87.3–96.3%) and negative predictive value (NPV) of 57.1% (45.5–68.7%) [Figure 1]. This result is similar to the recommended kit cut-off point (7.25 ng/µl). For FC our optimal cut-off value differed from that proposed by the manufacturer (148 mg/kg versus 50mg/kg, respectively), suggesting that FC levels are elevated in the AAD group. ROC analysis resulted in an AUC of 0.86 (0.81–0.92), producing sensitivity of 81.8% (75.8–87.8%), specificity of 76.5% (64.9–88.1%), whilst PPV and NPV were 91.5% (86.9–96.1%) and 57.4% (45.6–69.2%), respectively (Figure 1). There was a high degree of correlation between FC and FL (r^2^ = 0.74) consistent across all patients groups [Figure S1].

**Table 3:** Faecal lactoferrin levels in Clostridium difficile infection (CDI) cases versus Antibiotic-associated diarrhoea (AAD) controls.

Faecal lactoferrin (ng/ul)	CDI Cases (n = 164)	AAD Controls (n = 52)		
Median (IQR)	57.9 (11.4–177.5)	2.7 (0.7–7.8)		
Range (Min. – Max.)	1,838.5 (0.5–1,839.0)	203.4 (0.1–203.5)		
**Percentile distribution**	**CDI Cases (n = 164)**	**AAD Controls (n = 52)**	**Adjusted P-value** *	**Adjusted OR (95% CI)**
Low (Comparator group)	20	33	N/A	N/A
Medium	41	14	<0.0001	5.03 (2.05–12.34)
High	51	3	<0.0001	31.67 (8.14–123.26)
Very high	52	2	<0.0001	41.57 (8.55–202.10)
**Global p-value <1×10^−5^**				

*CDI: Clostridium difficile infection; AAD: Antibiotic-associated diarrhoea; n: number; IQR: Interquartile range; Min.: Minimum; Max.: Maximum; OR: Odds ratios; CI: Confidence interval; N/A: Not applicable.*

*P-value was calculated using binary logistic regression with data grouped into percentiles. Analysis was adjusted for significant covariates BMI and time delay between testing positive and subsequent recruitment.

**Table 4:** Faecal calprotectin levels in Clostridium difficile infection (CDI) cases versus Antibiotic-associated diarrhoea (AAD) controls.

Faecal calprotectin (mg/kg)	CDI Cases (n = 159)	AAD Controls (n = 51)		
Median (IQR)	684.8 (203.7–1,581.0)	66.5 (23.1–145.7)		
Range (Min – Max)	21,440.5 (9.7–21,450.2)	1,807.8 (3.1–1,811)		
**Percentile distribution**	**CDI Cases (n = 159)**	**AAD Controls (n = 51)**	**Adjusted P-value** *	**Adjusted OR (95% CI)**
Low (Comparator group)	21	31	N/A	N/A
Medium	38	15	0.02	3.03 (1.21–7.53)
High	49	4	<0.0001	21.82 (6.13–77.71)
Very high	51	1	<0.0001	85.87 (10.21–721.90)
**Global p-value <1×10^−5^**				

*CDI: Clostridium difficile infection; AAD: Antibiotic-associated diarrhoea; n: number; IQR: Interquartile range; Min.: Minimum; Max.: Maximum; OR: Odds ratios; CI: Confidence interval; N/A: Not applicable.*

*P-value was calculated using binary logistic regression with data grouped into percentiles. Analysis was adjusted for significant covariates BMI, score on Charlson Comorbidity Index (exclusive of age) and time delay between testing positive & subsequent recruitment.

**Figure 2:**
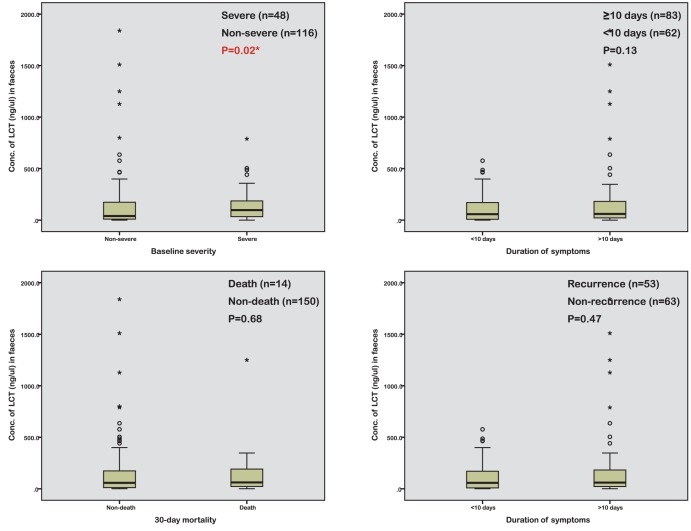
Boxplots for faecal lactoferrin concentrations in relation to *Clostridium difficile* infection outcomes. i) Severity at baseline (AUC = 0.60); ii) Prolonged symptoms (AUC = 0.56); iii) 30-day mortality (AUC = 0.53); and iv) 90-day recurrence (AUC = 0.55). Faecal lactoferrin was measured in 164 CDI cases. Data regarding duration of symptoms and disease recurrence was unavailable for 19 and 48 cases, respectively.

Sub-group percentile analysis identified that FL but not FC correlated with severe disease (FL: 104.6 vs. 40.1 ng/µl, p = 0.02; FC: 969.3 vs. 512.7 mg/kg, p = 0.09) [Figure 2; for FC please see Figure S2]. There was considerable overlap for FL levels between patients with severe and non-severe disease (Figure 2). For duration of symptoms, a significant association was observed with FC only when extreme percentiles were compared (p = 0.02), but this was not significant when all percentiles were included (p = 0.08). No significant associations were identified with the other outcome measures [Figure 2; for FC please see Figure S2]. Carriers of the ribotype 027 generally displayed higher levels of both faecal markers (median 1011 vs. 658 mg/kg, p = 0.09 for FC; median 83.2 versus 51.0 ng/µl, p = 0.57 for FL), but this was not significant. Median (range) levels of both FC and FL were higher in culture positive compared with culture negative samples but not significantly (712.2 (9.7–6,415.4) versus 345.8 (22.9–21,450.2) mg/kg, p = 0.46 for FC; 63.5 (0.5–1,839.0) vs. 31.7 (2.6–318.5) ng/µl, p = 0.22 for FL). Median levels of both FC and FL were however significantly higher in culture negative patients compared to AAD controls (345.8 versus 66.5 mg/kg, p<0.01 for FC; 31.7 versus 2.7 ng/µl, p<0.001 for FL).

## Discussion

FC and FL are derived from neutrophils in faecal material, and have been shown to correlate with the degree of inflammation in diseases such as IBD. Since CDI is also characterised histologically by intense neutrophilic infiltration [39], FC and FL may represent potential biomarkers of disease activity. Using a prospective cohort of inpatient CDI cases and AAD controls, we confirmed previous findings that both FC and FL increase during CDI (p<0.0001) [20], [23], [24], [26], [27]. There was a high degree of correlation between the two biomarkers, not surprising given their cellular origin. No previous CDI studies have evaluated both faecal biomarkers in the same patient group. These findings are consistent with those seen in IBD [12], [13], [15]–[18], [21].

There are more studies on FL than FC for CDI (Table 1) but only a few have provided quantitative data. For FL, the reported mean/median values for CDI cases have differed markedly across studies (33–961 µg/ml; Table 1) [20], [25], [26], [29]. Our median value is at the lower end of this range (57.9 ng/µl). By contrast, our observed median level for FC was markedly higher than that in the two previous CDI studies (648.8 mg/kg versus 192 and 249–336 mg/kg) [23], [24]. The median levels in our AAD controls were lower for both FL and FC (2.7 ng/µl versus 22.6–22.8 µg/ml and 66.5 mg/kg versus 106 µg/g, respectively; Table 1) than reported previously in the two FL studies and one FC study that included diarrhoea controls in their analysis [20], [24], [26]. Another study showed that 171 of 196 healthy controls (87%) had an FC level less than 15 mg/l [23], a similar observation to that seen in our AAD controls (41/51; 80%). Considerable variability was observed in different patients with CDI, which is consistent with data from IBD studies for both FL (4.34–179 µg/ml) [13], [16]–[18], [21], [40] and FC (164–2171 mg/kg) [13], [14], [16], [17], [19], [22], [40]–[43].

While our data show that FC and FL can differentiate between CDI and AAD, the use of these biomarkers for diagnosis per se would not add much value to the diagnostic paradigms currently in place. However, identification of patients with complicated CDI disease (for example disease leading to more prolonged symptoms and recurrent disease) would be useful. Our results show an association between FL levels and disease severity (p = 0.02) but not with FC (p = 0.09). This is an isolated finding, which taken together with the fact that there was a great deal of variability in actual concentrations, with significant overlap between the two groups (Figure 2), limits its clinical applicability. Furthermore, we observed no association with the other outcome measures evaluated. It is important to note that we had adequate statistical power to detect all of these outcomes except for 30-day mortality and FL and prolonged symptoms (Table S1).

Direct comparisons between this and other studies are limited by variability in methodologies adopted, the lack of quantitative data, and differences in the severity grading criteria [28]–[30]. Another problem may result from the potential short-lived characteristics of the biomarkers, which may hamper the predictive power of these markers unless they are captured within specific timeframes. A longitudinal study of FL [44] suggested that FL could be used to monitor disease activity and response since FL tends to return to baseline very rapidly following remission [29], [44].

Our study has limitations. Firstly, we only used a single laboratory test (ELISA for CDT) for the primary identification of CDI cases. Although this is still a common procedure, modern algorithms currently make use of a more sensitive first step screening - based on either GDH, or NAAT - to minimise the odds of reporting false negative results. Therefore it is possible that our cohort may have lacked a fully representative range of cases. Furthermore, our AAD controls were not a homogenous group of patients and it is difficult to assess their fitness for this sort of analysis given that antimicrobials and/or PPIs may not be the sole underlying cause of their gastrointestinal tract dysbiosis.

Nevertheless, our data highlight the difficulties in using FL and FC as biomarkers for CDI. The variability observed would reduce predictive accuracy, and cannot be completely ascribed to variations in disease severity. Part of the variability may be due to differences in laboratory methodology. The volume of diluent for specimen suspension, and laboratory handling can each influence results, and caution should be exercised in the interpretation of single results [45]. Although serial testing may have some value, it would add to the cost, and may be challenging in diseases such as CDI, thus further reducing its utility. Furthermore, these biomarkers can be elevated due to other diseases [45], and this is particularly important for CDI where infected patients are usually elderly with multiple co-morbidities.

There are no guidelines concerning the use of faecal biomarkers for the classification of CDI cases. In IBD research, where faecal biomarkers constitute a potential non-invasive alternative to colonoscopy, the most recent diagnostics guidance by UK National Institute for Health and Care Excellence (NICE) [46] still recommends that further research is needed on the use and clinical utility of faecal marker testing. Biomarkers which can act as indicators of disease, disease relapse and disease stratification, are also needed to direct CDI therapies more effectively. Our results suggest that FC and FL have limited applicability in this role.

## Supporting Information

Figure S1 **Correlation plot of Faecal Lactoferrin and Faecal Calprotectin concentrations in all patients (cases and controls combined; n = 210).**
(TIF)

Figure S2 **Boxplots for faecal calprotectin concentrations in relation to** ***Clostridium difficile*** **infection outcomes.** i) Severity at baseline (AUC = 0.59); ii) Prolonged symptoms (AUC = 0.58); iii) 30-day mortality (AUC = 0.49); and iv) 90-day recurrence (AUC = 0.58). Faecal calprotectin was measured in 159 CDI cases. Data regarding duration of symptoms and disease recurrence was unavailable for 18 and 47 cases, respectively.
(TIF)

Table S1 **Assessment of power across** ***Clostridium difficile*** **infection outcome analyses.** *a: To achieve 80% power we would require 749 patients in both sample groups. b: To achieve 80% power we would require 1370 patients in both sample groups. c: To achieve 80% power we would require 167 patients in both sample groups.*
(DOCX)
