# Supplementary figures and images for: Calprotectin and Lactoferrin Faecal Levels in Patients with Clostridium difficile Infection (CDI): A Prospective Cohort Study
Source: PLoS One. 2014 Aug 29;9(8):e106118. doi: 10.1371/journal.pone.0106118 (PMC4149523; doi:10.1371/journal.pone.0106118)

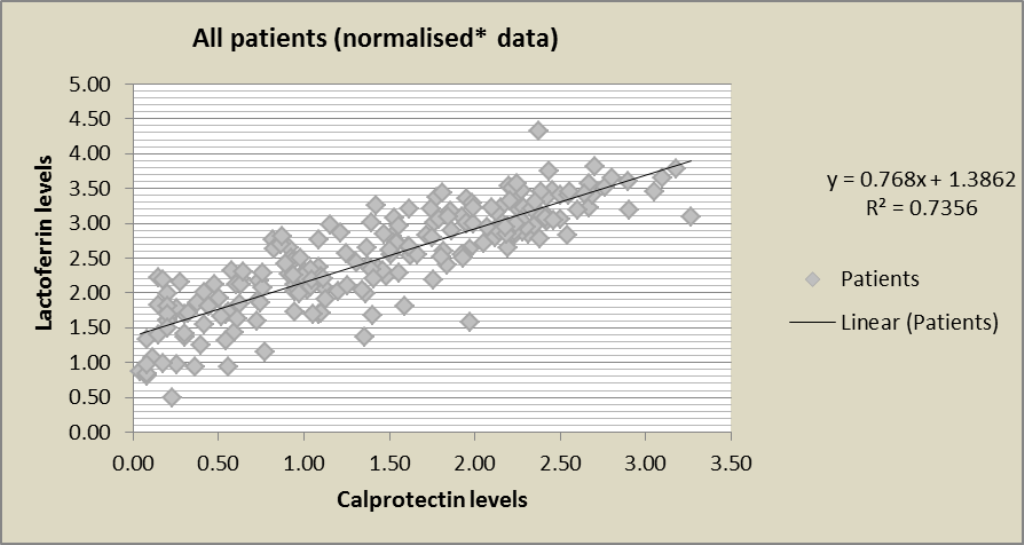

Supplement: Figure S1 — Correlation plot of Faecal Lactoferrin and Faecal Calprotectin concentrations in all patients (cases and controls combined; n = 210). (TIF) [file pone.0106118.s001.tif]

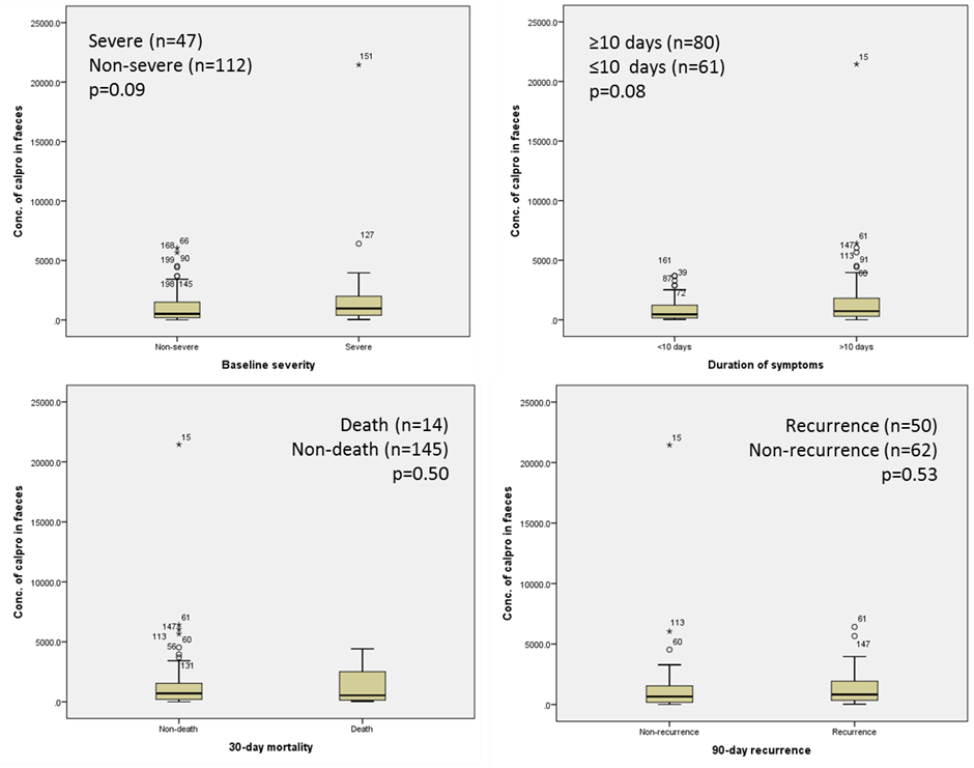

Supplement: Figure S2 — Boxplots for faecal calprotectin concentrations in relation to Clostridium difficile infection outcomes. i) Severity at baseline (AUC = 0.59); ii) Prolonged symptoms (AUC = 0.58); iii) 30-day mortality (AUC = 0.49); and iv) 90-day recurrence (AUC = 0.58). Faecal calprotectin was measured in 159 CDI cases. Data regarding duration of symptoms and disease recurrence was unavailable for 18 and 47 cases, respectively. (TIF) [file pone.0106118.s002.tif]
