# Supplementary material for: Calprotectin and Lactoferrin Faecal Levels in Patients with Clostridium difficile Infection (CDI): A Prospective Cohort Study
Source: PLoS One. 2014 Aug 29;9(8):e106118. doi: 10.1371/journal.pone.0106118 (PMC4149523; doi:10.1371/journal.pone.0106118)
Supplement: Table S1 — Assessment of power across Clostridium difficile infection outcome analyses. a: To achieve 80% power we would require 749 patients in both sample groups. b: To achieve 80% power we would require 1370 patients in both sample groups. c: To achieve 80% power we would require 167 patients in both sample groups. (DOCX) [file pone.0106118.s003.docx]

**Table S1 – Assessment of power across *Clostridium difficile* infection outcome analyses**

| **Disease outcome** | **Power (%)** | |
| --- | --- | --- |
|  | Faecal lactoferrin | Faecal calprotectin |
| Case versus control | 99 | 99 |
| Prolonged symptoms | 13^a^ | 99 |
| 30-day mortality | 6^b^ | 19^c^ |
| 90-day recurrence | 99 | 97 |
| Disease severity at baseline | 99 | 99 |
